# Supplementary material for: TIM-3 Expression and M2 Polarization of Macrophages in the TGFβ-Activated Tumor Microenvironment in Colorectal Cancer
Source: Cancers (Basel). 2023 Oct 11;15(20):4943. doi: 10.3390/cancers15204943 (PMC10605063; doi:10.3390/cancers15204943)
Supplement: Supplementary file 1 [file cancers-15-04943-s001.zip › Figures S1-S6.pptx]

## Slide 1
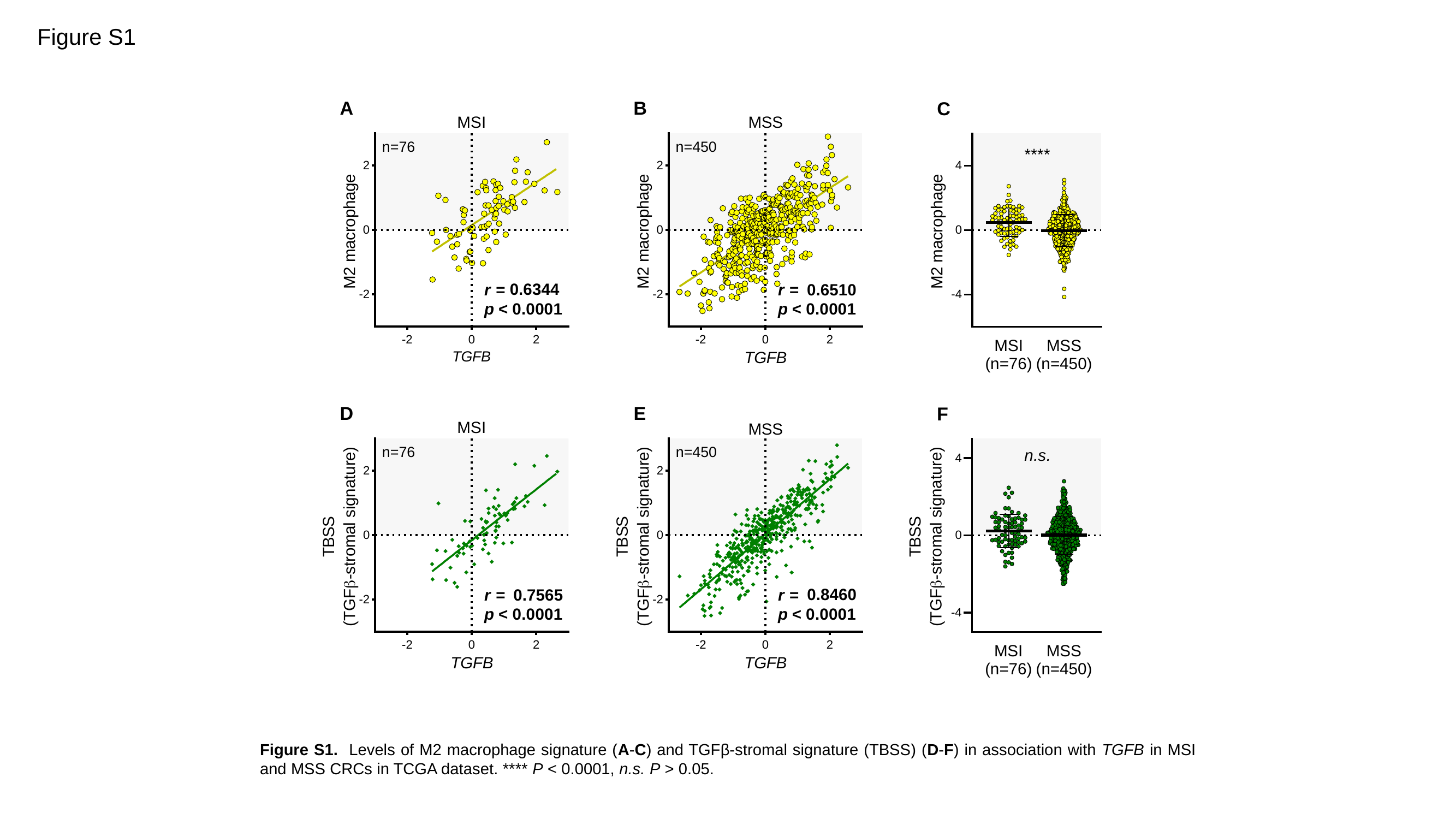

Figure S1
Figure S1. Levels of M2 macrophage signature (A-C) and TGFβ-stromal signature (TBSS) (D-F) in association with TGFB in MSI and MSS CRCs in TCGA dataset. **** P < 0.0001, n.s. P > 0.05.

## Slide 2
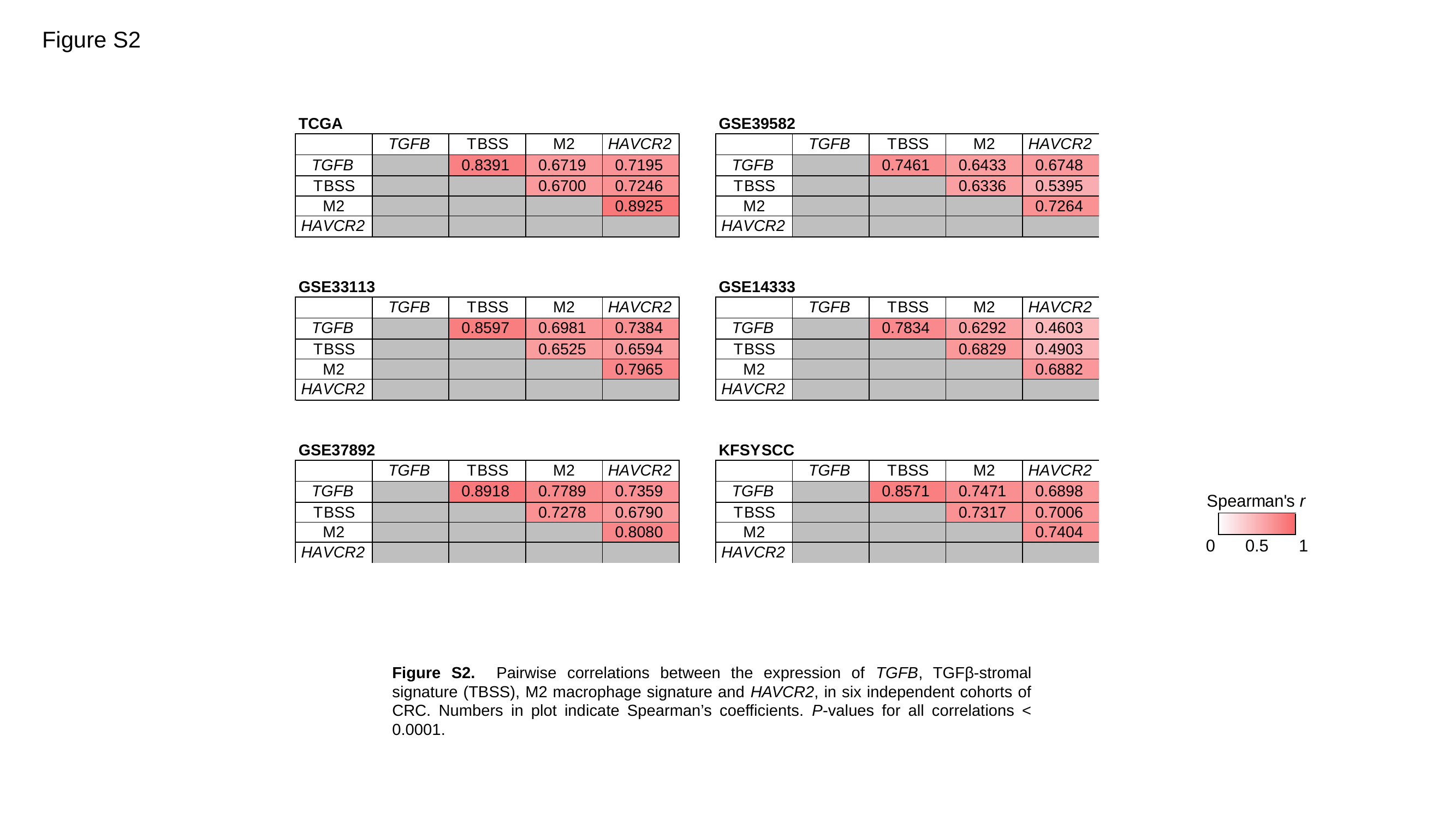

Figure S2
Figure S2. Pairwise correlations between the expression of TGFB, TGFβ-stromal signature (TBSS), M2 macrophage signature and HAVCR2, in six independent cohorts of CRC. Numbers in plot indicate Spearman’s coefficients. P-values for all correlations < 0.0001.

## Slide 3
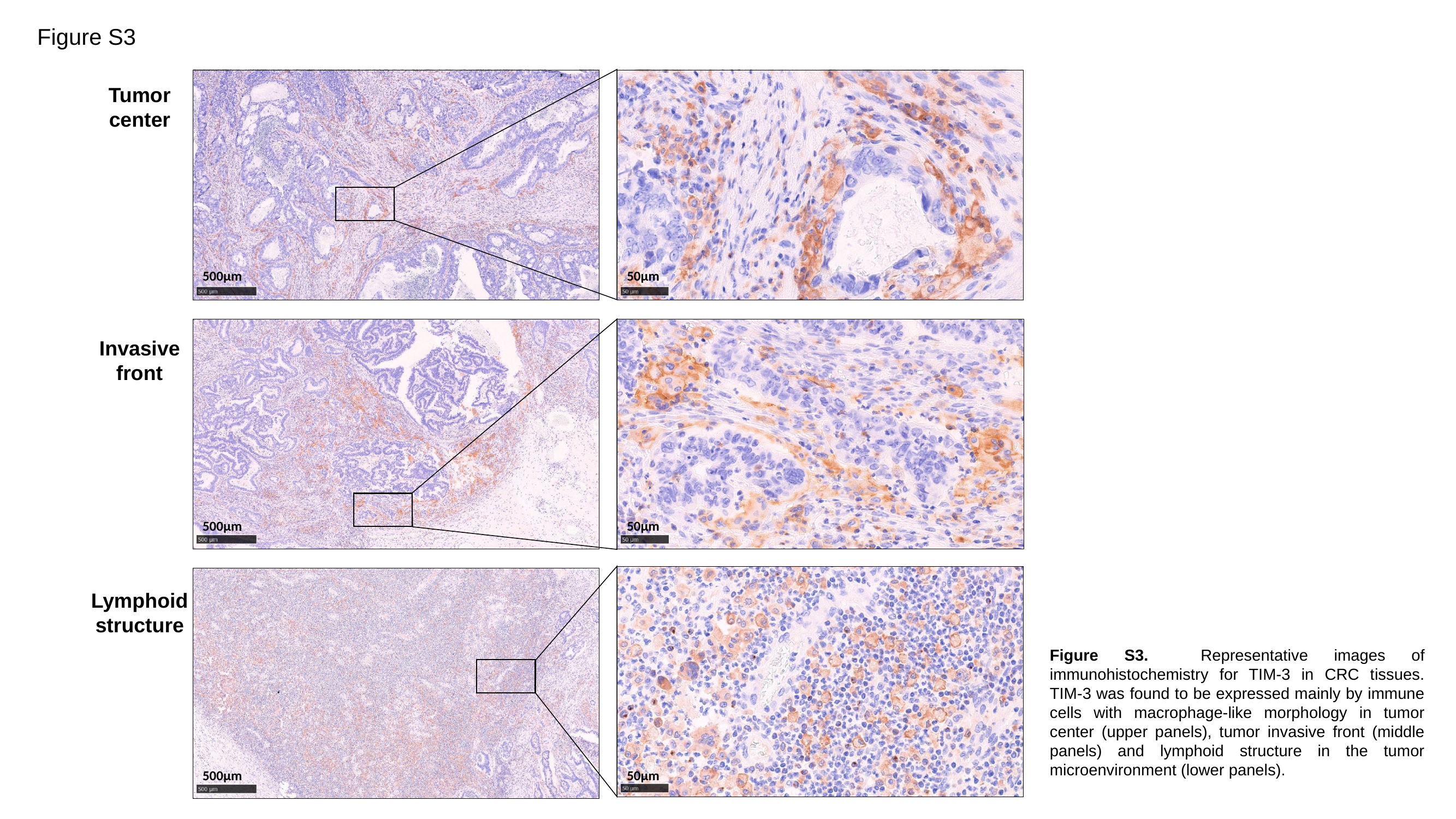

Figure S3
Tumor center
500µm
50µm
Invasive front
500µm
50µm
Lymphoid structure
Figure S3. Representative images of immunohistochemistry for TIM-3 in CRC tissues. TIM-3 was found to be expressed mainly by immune cells with macrophage-like morphology in tumor center (upper panels), tumor invasive front (middle panels) and lymphoid structure in the tumor microenvironment (lower panels).
500µm
50µm

## Slide 4
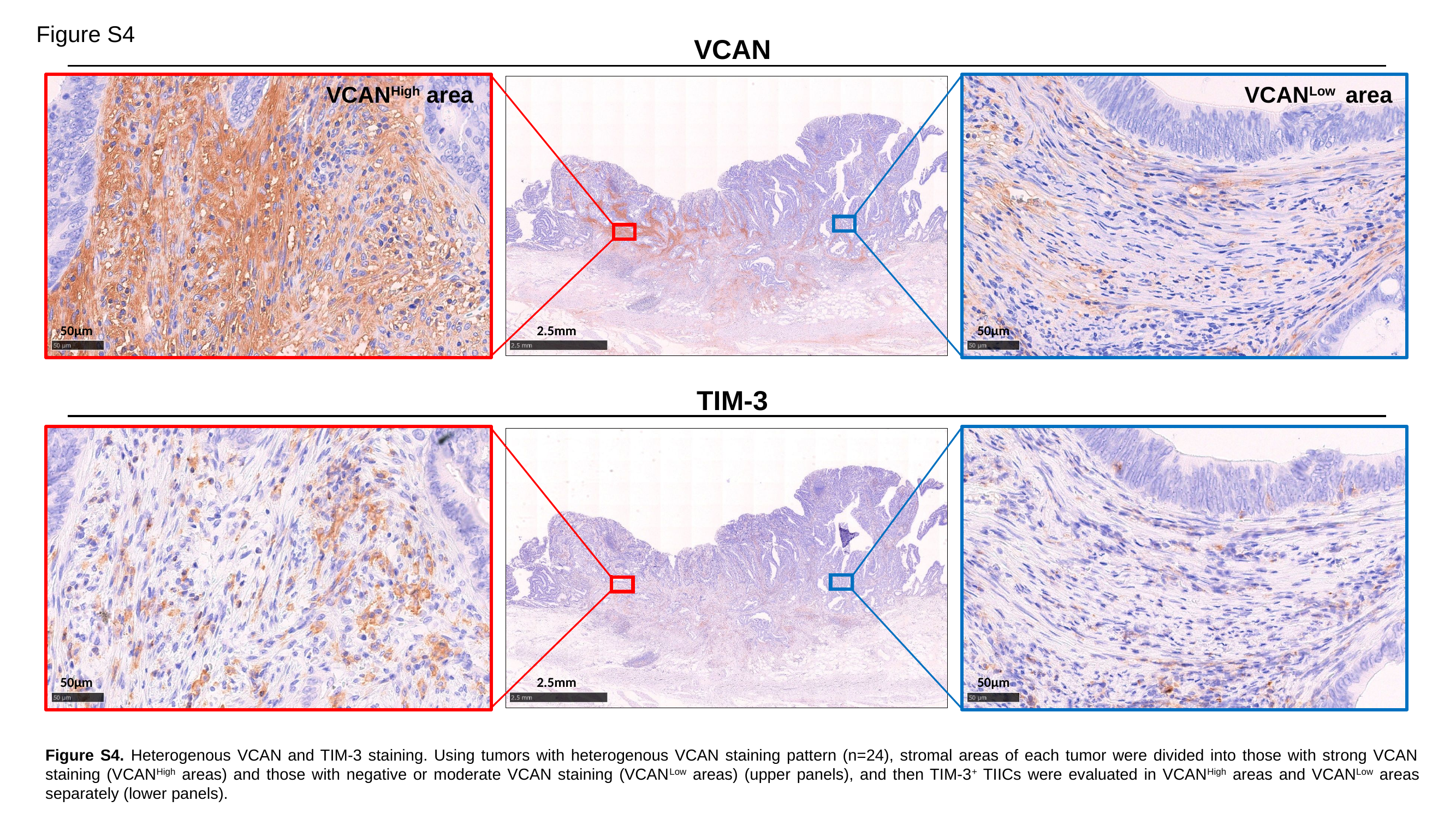

Figure S4
VCAN
VCANHigh area
VCANLow area
50µm
2.5mm
50µm
TIM-3
50µm
2.5mm
50µm
Figure S4. Heterogenous VCAN and TIM-3 staining. Using tumors with heterogenous VCAN staining pattern (n=24), stromal areas of each tumor were divided into those with strong VCAN staining (VCANHigh areas) and those with negative or moderate VCAN staining (VCANLow areas) (upper panels), and then TIM-3+ TIICs were evaluated in VCANHigh areas and VCANLow areas separately (lower panels).

## Slide 5
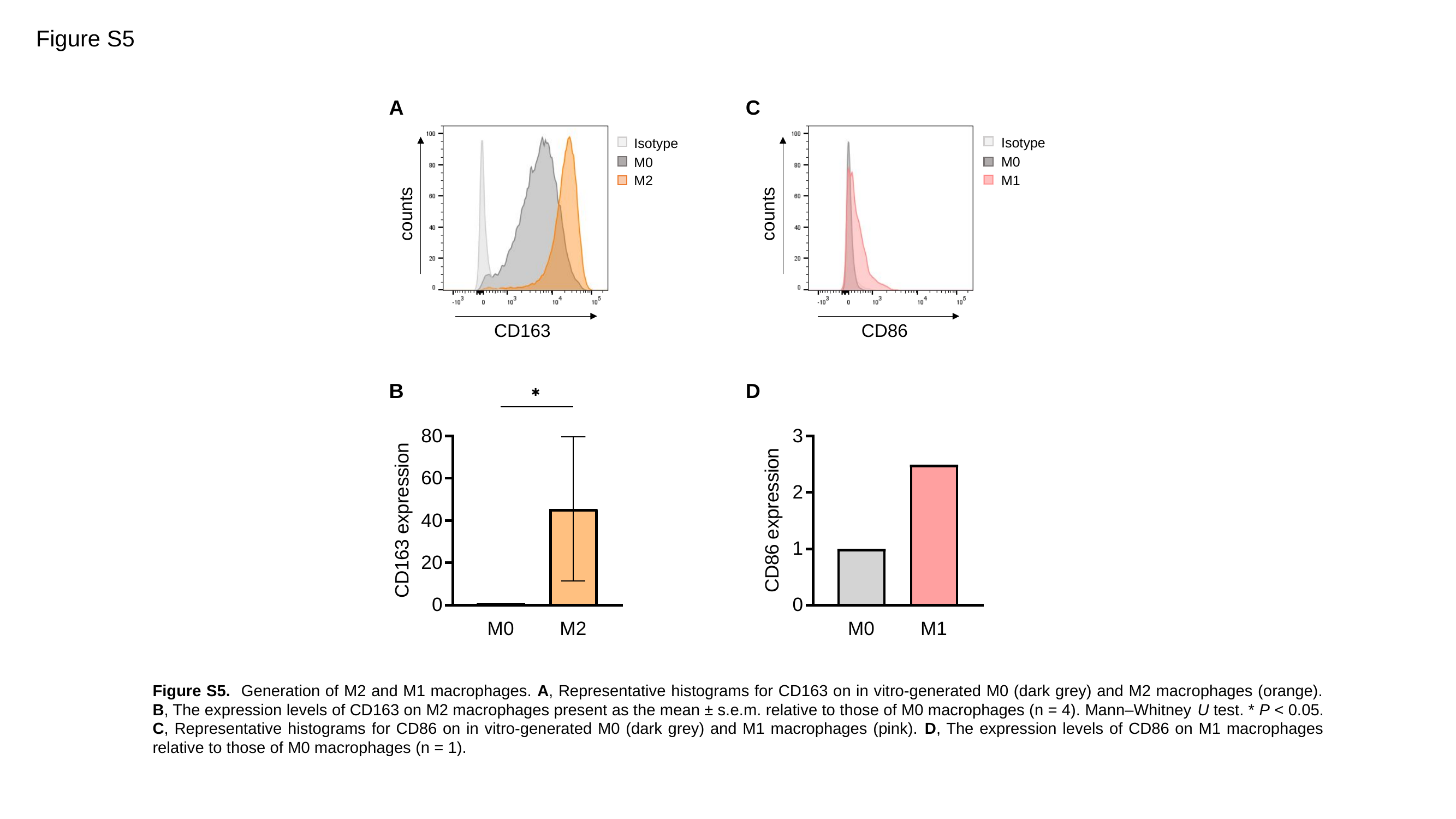

Figure S5
A
C
Isotype
M0
M1
Isotype
M0
M2
counts
counts
CD163
CD86
B
D
Figure S5. Generation of M2 and M1 macrophages. A, Representative histograms for CD163 on in vitro-generated M0 (dark grey) and M2 macrophages (orange). B, The expression levels of CD163 on M2 macrophages present as the mean ± s.e.m. relative to those of M0 macrophages (n = 4). Mann–Whitney U test. * P < 0.05. C, Representative histograms for CD86 on in vitro-generated M0 (dark grey) and M1 macrophages (pink). D, The expression levels of CD86 on M1 macrophages relative to those of M0 macrophages (n = 1).

## Slide 6
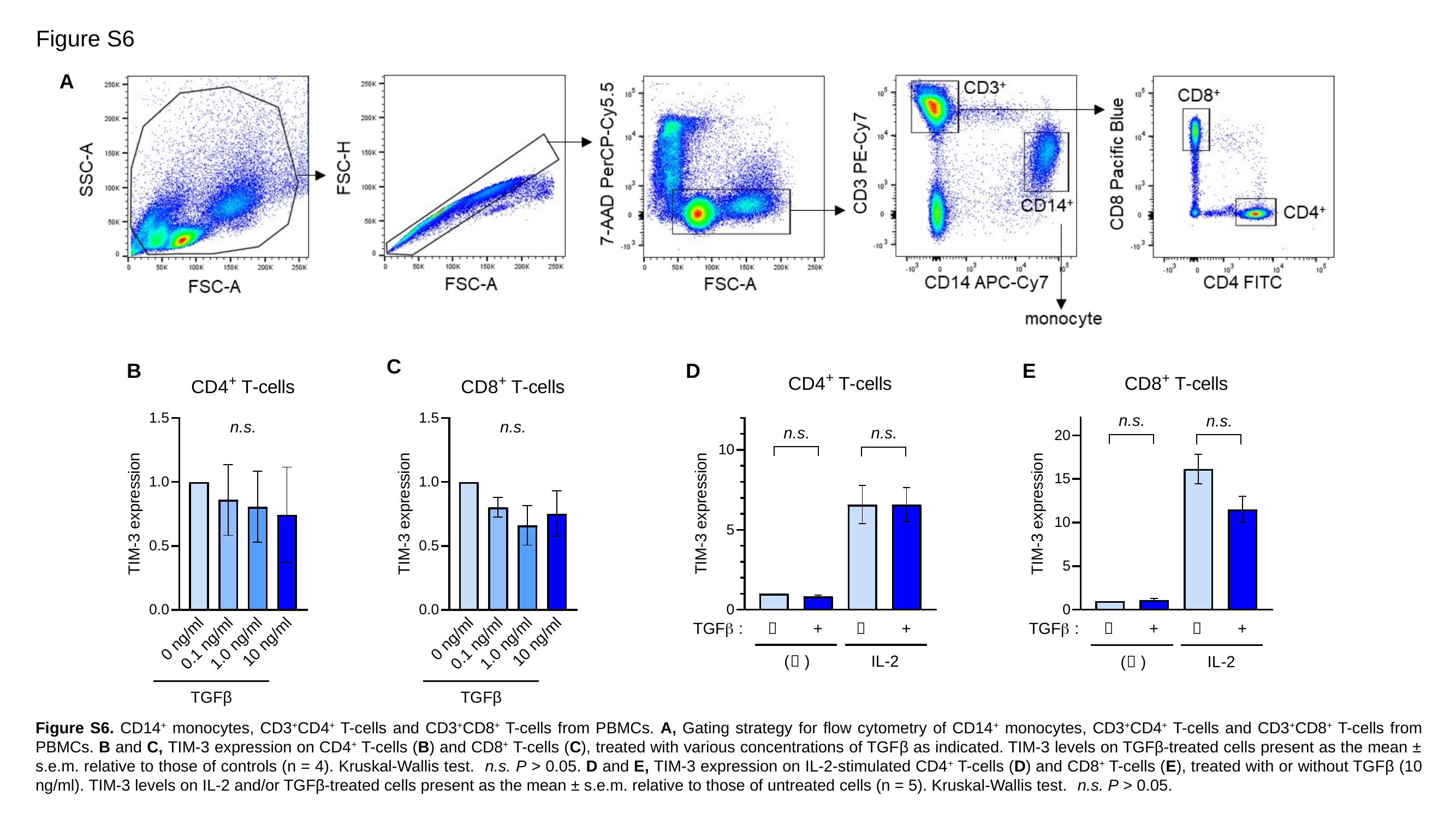

Figure S6
A
C
E
B
D
Figure S6. CD14+ monocytes, CD3+CD4+ T-cells and CD3+CD8+ T-cells from PBMCs. A, Gating strategy for flow cytometry of CD14+ monocytes, CD3+CD4+ T-cells and CD3+CD8+ T-cells from PBMCs. B and C, TIM-3 expression on CD4+ T-cells (B) and CD8+ T-cells (C), treated with various concentrations of TGFβ as indicated. TIM-3 levels on TGFβ-treated cells present as the mean ± s.e.m. relative to those of controls (n = 4). Kruskal-Wallis test. n.s. P > 0.05. D and E, TIM-3 expression on IL-2-stimulated CD4+ T-cells (D) and CD8+ T-cells (E), treated with or without TGFβ (10 ng/ml). TIM-3 levels on IL-2 and/or TGFβ-treated cells present as the mean ± s.e.m. relative to those of untreated cells (n = 5). Kruskal-Wallis test. n.s. P > 0.05.
