# Supplementary material for: TIM-3 Expression and M2 Polarization of Macrophages in the TGFβ-Activated Tumor Microenvironment in Colorectal Cancer
Source: Cancers (Basel). 2023 Oct 11;15(20):4943. doi: 10.3390/cancers15204943 (PMC10605063; doi:10.3390/cancers15204943)
Supplement: Supplementary file 1 [file cancers-15-04943-s001.zip › Table S1.pdf]

Table S1. Datasets used in this study.

Datasets	Data sources	Number of samples (total, n=2240)	Platforms	References
TCGA	TCGA/ cBioPortal/ LinkedOmics	592	Illumina HiSeq	(1-3)
GSE39582	GEO	566	Affymetrix U133+2.0	(4)
GSE33113	GEO	90	Affymetrix U133+2.0	(5)
GSE14333	GEO	290	Affymetrix U133+2.0	(6)
GSE37892	GEO	130	Affymetrix U133+2.0	(7)
KFSYSCC	Synapse	307	Affymetrix U133+2.0	(8,9)
GSE171682	GEO	174 (87 CRCs, 87 CRC organoids)	Illumina HiSeq 2500	(10)
GSE100550	GEO	91 (21 CRCs, 18 CRC organoids, 34 CRC xenografts, 18 CRC cell lines)	Affymetrix HT HG-U133+ PM	(11)

References for Supplementary Table

1. Cancer Genome Atlas N. Comprehensive molecular characterization of human colon and rectal cancer. *Nature* 2012;**487**(7407):330-7 doi 10.1038/nature11252.

2. Gao J, Aksoy BA, Dogrusoz U, Dresdner G, Gross B, Sumer SO*, et al.* Integrative analysis of complex cancer genomics and clinical profiles using the cBioPortal. *Sci Signal* 2013;**6**(269):pl1 doi 10.1126/scisignal.2004088.

3. Vasaikar SV, Straub P, Wang J, Zhang B. LinkedOmics: analyzing multi-omics data within and across 32 cancer types. *Nucleic Acids Res* 2018;**46**(D1):D956-D63 doi 10.1093/nar/gkx1090.

4. Marisa L, de Reyniès A, Duval A, Selves J, Gaub MP, Vescovo L*, et al.* Gene expression classification of colon cancer into molecular subtypes: characterization, validation, and prognostic value. *PLoS Med* 2013;**10**(5):e1001453 doi 10.1371/journal.pmed.1001453.

5. de Sousa E Melo F, Colak S, Buikhuisen J, Koster J, Cameron K, de Jong JH*, et al.* Methylation of cancer-stem-cell-associated Wnt target genes predicts poor prognosis in colorectal cancer patients. *Cell Stem*

Cell 2011;**9**(5):476-85 doi 10.1016/j.stem.2011.10.008.

6. Jorissen RN, Gibbs P, Christie M, Prakash S, Lipton L, Desai J*, et al.* Metastasis-Associated Gene Expression Changes Predict Poor Outcomes in Patients with Dukes Stage B and C Colorectal Cancer. *Clin Cancer Res* 2009;**15**(24):7642-51 doi 10.1158/1078-0432.CCR-09-1431.
7. Laibe S, Lagarde A, Ferrari A, Monges G, Birnbaum D, Olschwang S*, et al.* A seven-gene signature aggregates a subgroup of stage II colon cancers with stage III. *OMICS* 2012;**16**(10):560-5 doi 10.1089/omi.2012.0039.
8. Guinney J, Ferte C, Dry J, McEwen R, Manceau G, Kao KJ*, et al.* Modeling RAS phenotype in colorectal cancer uncovers novel molecular traits of RAS dependency and improves prediction of response to targeted agents in patients. *Clin Cancer Res* 2014;**20**(1):265-72 doi 10.1158/1078-0432.CCR-13-1943.
9. Lal N, White BS, Goussous G, Pickles O, Mason MJ, Beggs AD*, et al.* KRAS Mutation and Consensus Molecular Subtypes 2 and 3 Are Independently Associated with Reduced Immune Infiltration and Reactivity in Colorectal Cancer. *Clin Cancer Res* 2018;**24**(1):224-33 doi 10.1158/1078-0432.CCR-17-1090.
10. Cho EJ, Kim M, Jo D, Kim J, Oh JH, Chung HC*, et al.* Immuno-genomic classification of colorectal cancer organoids reveals cancer cells with intrinsic immunogenic properties associated with patient survival. *J Exp Clin Cancer Res* 2021;**40**(1):230 doi 10.1186/s13046-021-02034-1.
11. Linnekamp JF, Hooff SRV, Prasetyanti PR, Kandimalla R, Buikhuisen JY, Fessler E*, et al.* Consensus molecular subtypes of colorectal cancer are recapitulated in in vitro and in vivo models. *Cell Death Differ* 2018;**25**(3):616-33 doi 10.1038/s41418-017-0011-5.
